# Association between Medicare’s Hospital Readmission Reduction Program and readmission rates across hospitals by medicare bed share

**DOI:** 10.1186/s12913-021-06253-2

**Published:** 2021-03-19

**Authors:** Souvik Banerjee, Michael K. Paasche-Orlow, Danny McCormick, Meng-Yun Lin, Amresh D. Hanchate

**Affiliations:** 1 Department of Humanities and Social Sciences, Indian Institute of Technology Bombay, Mumbai, Maharashtra India; 2 General Internal Medicine, Boston University School of Medicine, Boston, MA USA; 3 Division of Social and Community Medicine, Department of Medicine, Harvard Medical School, Cambridge Health Alliance, Cambridge, MA USA; 4 Department of Social Sciences and Health Policy, Wake Forest School of Medicine, Medical Center Boulevard, Winston-Salem, NC 27157-1063 USA

**Keywords:** Readmissions, Hospital performance-based penalty, Medicare bed share

## Abstract

**Background:**

Medicare’s Hospital Readmissions Reduction Program (HRRP), implemented beginning in 2013, seeks to incentivize Inpatient Prospective Payment System (IPPS) hospitals to reduce 30-day readmissions for selected inpatient cohorts including acute myocardial infarction, heart failure, and pneumonia. Performance-based penalties, which take the form of a percentage reduction in Medicare reimbursement for all inpatient care services, have a risk of unintended financial burden on hospitals that care for a larger proportion of Medicare patients. To examine the role of this unintended risk on 30-day readmissions, we estimated the association between the extent of their Medicare share of total hospital bed days and changes in 30-day readmissions.

**Methods:**

We used publicly available nationwide hospital level data for 2009–2016 from the Centers for Medicare and Medicaid Services (CMS) Hospital Compare program, CMS Final Impact Rule, and the American Hospital Association Annual Survey. Using a quasi-experimental difference-in-differences approach, we compared pre- vs. post-HRRP changes in 30-day readmission rate in hospitals with high and moderate Medicare share of total hospital bed days (“Medicare bed share”) vs. low Medicare bed share hospitals.

**Results:**

We grouped the 1904 study hospitals into tertiles (low, moderate and high) by Medicare bed share; the average bed share in the three tertile groups was 31.2, 47.8 and 59.9%, respectively. Compared to low Medicare bed share hospitals, high bed share hospitals were more likely to be non-profit, have smaller bed size and less likely to be a teaching hospital. High bed share hospitals were more likely to be in rural and non-large-urban areas, have fewer lower income patients and have a less complex patient case-mix profile. At baseline, the average readmissions rate in the low Medicare bed share (control) hospitals was 20.0% (AMI), 24.7% (HF) and 18.4% (pneumonia). The observed pre- to post-program change in the control hospitals was − 1.35% (AMI), − 1.02% (HF) and − 0.35% (pneumonia). Difference in differences model estimates indicated no differential change in readmissions among moderate and high Medicare bed share hospitals.

**Conclusions:**

HRRP penalties were not associated with any change in readmissions rate. The CMS should consider alternative options – including working collaboratively with hospitals – to reduce readmissions.

**Supplementary Information:**

The online version contains supplementary material available at 10.1186/s12913-021-06253-2.

## Background

Under the aegis of the Patient Protection and Affordable Care Act, the Hospital Readmission Reduction Program (HRRP), enacted in 2010 and implemented beginning in 2013, was part of a broader goal of incentivizing improvement in quality of inpatient care, by linking Medicare reimbursements to a hospital with its relative performance on readmissions of patients hospitalized for selected conditions. The HRRP program imposed financial penalties in the form of reduced reimbursement for all inpatient claims from hospitals having “higher-than-expected” readmission rates for patients hospitalized for selected acute admission conditions. In the initial two years after implementation (FY 2013 and 2014), the admission conditions considered were acute myocardial infarction (AMI), heart failure, and pneumonia; admissions for chronic obstructive pulmonary disease and elective primary total hip and/or knee arthroplasty were included in FY 2015, and for coronary artery bypass graft in FY 2017. The premise of the penalties was that high readmission rates were an indicator of deficiencies in discharge protocols and other processes of care, and more generally a marker of poor quality of care provided by hospitals [1]. Penalties were levied on hospitals beginning in October 2012 (FY 2013), with a maximum penalty of 1%; it increased to 3% in FY 2015 and has remained at this level since then. In addition, since penalties applied to all inpatient care provided by a hospital to Medicare patients, the size of the HRRP penalty for any given hospital is determined not only by performance on readmissions but also by the share of inpatient admissions for which Medicare was the payer. Thereby, the program included an unintended additional penalty burden for hospitals serving more Medicare patients. This unintended burden is, in principle, unaffected by the revisions beginning in FY 2019, wherein comparisons are among hospitals with similar share of socioeconomically vulnerable patients (measured by patients with Medicaid eligibility) [2].

While there is a growing literature on changes in readmission rates following HRRP [3–11], there is limited work on the role of the unintended penalty burden as an added motivation to improve readmission performance. Using New York state data based on the first year (2013) of program experience, one study found a secular reduction in risk-adjusted readmissions across all state hospitals, with similar reductions in hospitals with greater financial reliance on Medicare patients, as measured by the share of bed days for Medicare payments out of total bed days [12]. No studies have examined the experience in other states. Further, evidence since 2013 indicates a trend of growing financial burden from penalties. Maximum penalty rates increased after 2013, further increasing the potential financial burden. Actual average penalty rates also increased from 0.29% in 2013 to 0.60% in 2017 [13]. Frequency of repeated penalties over multiple years is high, with 52% of participating hospitals experiencing a penalty in every year during 2013–2017 [13].

In light of the increased financial penalties at stake, and evidence of declining readmission rates, we used national data from 2009 to 2016 to examine if hospitals with higher financial reliance on Medicare payments demonstrated a larger decrease in readmission rates.

## Methods

### Data sources

We used data from the Centers for Medicare and Medicaid Services (CMS) Hospital Compare program (Department of Health and Human Services) from 2009 to 2016 [14], CMS Final Impact Rule (CMS.gov) over the same period [15], and data from the American Hospital Association Annual Survey from 2009 [16]. Hospitals that were not paid under the Inpatient Prospective Payment System (IPPS) system were excluded from our analysis; excluded hospitals were those in the state of Maryland, critical access hospitals, pediatric hospitals, long-term care facilities, rehabilitation hospitals, psychiatric hospitals, and Veterans Affairs hospitals [17]. In addition, we excluded hospitals that were not included in the CMS Hospital Compare reporting of 30-day risk-adjusted readmission rates for each year during the study period [18]. This study included all IPPS hospitals over the period 2009–2016; there were 2756 IPPS hospitals in 2009 and 2607 IPPS hospitals in 2016.

### Readmission outcomes

The analytic data consisted of longitudinal annual observations for the included IPPS hospitals over the period 2009–2016. The study outcomes were 30-day risk-adjusted readmission rates for AMI, heart failure, and pneumonia. Every year, the Hospital Compare Program reports 30-day risk-adjusted readmission rates for each hospital for each condition based on eligible admissions in the previous three years; differences between hospitals in patient characteristics, including age, sex, comorbid health conditions are adjusted for, as well as other unobserved, systematic hospital effects [19]. The pneumonia cohort observations were restricted to 2009–2015, since the CMS’ definition of pneumonia for this measure was modified in 2016, resulting in a large increase in the number of eligible admissions [20].

### Independent variables

The primary predictor of interest was *Medicare bed share*, defined as the share of total inpatient days of care provided to Medicare-reimbursed patients out of total inpatient days for all patients, and was grouped into tertiles (tertile 3 [“high”], tertile 2 [“moderate”] and tertile 1 [“low”]). In the absence of well-defined guidelines for grouping hospitals by Medicare bed share, we examined alternate categorizations including grouping by quartiles and using Medicare bed share as a continuous measure [12]. As the findings based on tertile and quartile categorizations were similar, we have reported findings based on the tertile grouping as our preferred specification; estimates from alternative groupings are reported in the Additional file 1. We hypothesized that high Medicare bed share hospitals had the greatest incentives to respond to the risk of HRRP penalties, followed by moderate and low Medicare bed share hospitals.

### Hospital characteristics

We identified several hospital characteristics in 2009 (baseline) as potential covariates on readmissions performance: bed size (fewer than 100, 100–199, and 200 or more); teaching hospital status indicated by membership in the Council of Teaching Hospitals; ownership (not-for-profit, government non-federal, and for-profit); region (northeast, midwest, south, and west); rural/urban location (large urban area, other urban area and rural area); share of low income patients (low, moderate and high) and hospital tertiles based on the complexity of patient case-mix.. Share of low income patients was based on the disproportionate share hospital patient percentage measure based on shares of patients covered by Medicaid or receiving supplementary Social Security payments [21]. Hospital-level case-mix is based on the average diagnostic related group (DRG) score, with higher score denoting patients with more complications or requiring more resource use during the inpatient stay [22].

### Analysis

We compared the characteristics of high vs. low and moderate vs. low Medicare bed share hospitals in 2009, the baseline year. T-tests were performed for differences between the different groups for continuous variables and chi-square tests for categorical variables. To explore the relationship between 30-day readmissions and Medicare bed share – treated as a continuous variable – a non-parametric locally weighted polynomial regression curve was fitted based on an unadjusted regression model predicting readmissions as a function of Medicare bed share [23]. We also plotted the 30-day readmission rates for each condition stratified by Medicare bed share tertiles from 2009 to 2016 to assess trends in the readmission outcome. Linear time series models were estimated to capture the average annual change in readmission rates for the same conditions over the study period [24].

The association between the size of the HRRP program incentives and readmission rates were estimated using a quasi-experimental difference-in-differences design, wherein pre- vs. post-HRRP changes in the outcome was compared between high vs. low Medicare bed share and between moderate vs. low Medicare bed share hospitals [25–27]. Since the HRRP program was announced in March 2010, we considered hospital readmissions in and after 2010 as potentially influenced by the HRRP program (i.e., post-HRRP). To this end, we defined a dichotomous indicator for pre- vs. post-HRRP (1 for observations between 2010 and 2016, 0 for observations in 2009).

To estimate the change in readmissions associated with HRRP across hospitals by Medicare bed share, we used a hospital-level linear random effects model with a difference-in-differences specification (Eq. 1) [25, 28].

1 $$ {\displaystyle \begin{array}{l}{\mathrm{READM}}_{\mathrm{it}}={\beta}_0+{\beta}_1\ast \mathrm{BEDSH}{2}_{\mathrm{it}}+{\beta}_2\ast \mathrm{BEDSH}{3}_{\mathrm{it}}+{\beta}_3\ast {\mathrm{POST}}_{\mathrm{it}}+{\beta}_4\ast \mathrm{BEDSH}{2}_{\mathrm{it}}\ast {\mathrm{POST}}_{\mathrm{it}}+\\ {}{\beta}_5\ast \mathrm{BEDSH}{3}_{\mathrm{it}}\ast {\mathrm{POST}}_{\mathrm{it}}+{\beta}_6\ast {\mathrm{OTHERCOV}}_{\mathrm{it}}+{u}_i+{e}_{\mathrm{it}}\end{array}} $$

*READM*_*it*_ denotes the readmission rate for hospital *i* in year *t*, *BEDSH*2 and *BEDSH*3 are indicators (0/1) of moderate and high Medicare bed share hospitals, *POST* indicates (0/1) the post-HRRP period and *OTHERCOV* denotes other model covariates of hospital characteristics in 2009 (e.g., hospital bed size, ownership) and year indicators. The change in readmissions associated with HRRP is given by *β*_4_ and *β*_5_ for moderate and high Medicare bed share hospitals, respectively. *u*_*i*_ is a random variable of unobserved systematic hospital-level differences in readmission rates. We obtained robust standard errors clustered at the hospital level [27, 29, 30]. The validity of the difference-in-differences approach depends on the similarity in pre-HRRP trends in the outcome measures across the Medicare bed share categories (“parallel trends assumption”) [26]. Due to availability of only one pre-HRRP data point, our test was based on comparison of the changes between 2009 and 2010 across Medicare bed share hospitals, excluding post-2010 data; these results are reported in Additional file 1. We performed a variety of sensitivity analyses relating to alternative grouping of Medicare bed share hospitals, choice of post-HRRP period (2011–2016; 2012–2016) and hospital-level fixed effects (in contrast to a random effects) specification [25, 31].

We performed all statistical analyses using Stata version 16.1 [32]. The institutional review board at the corresponding author’s affiliated institution considered this study exempt from human subjects review as no person-level data was involved.

## Results

The number of hospitals included in our analytic sample was 1904 for each year over the study period. The characteristics of the hospitals were compared in the baseline year, 2009, stratified by Medicare bed share tertiles in Table 1. The mean share of Medicare bed days was 59.9% for high, 47.8% for moderate, and 31.2% for low Medicare bed share hospitals. Higher Medicare bed share hospitals were more likely to have smaller bed size, be located in rural and non-large urban areas, have fewer low income patients and fewer patients with more complex case-mix.

**Table 1:** Hospital characteristics in baseline year (2009) by Medicare bed share tertiles

	High Medicare bed share (tertile 3) hospitals		*p* value	Moderate Medicare bed share (tertile 2) hospitals		p value	Low Medicare bed share (tertile 1) hospitals	
	(*N* = 634)			(*N* = 635)			(N = 635)	
	n	(%)		n	(%)		n	(%)
Medicare share inpatient days								
Mean	0.599		< 0.001	0.478		< 0.001	0.312	
Standard deviation	0.05			0.04			0.08	
Teaching hospital (COTH), n (%)	11	1.7	< 0.001	62	9.8	< 0.001	168	26.5
Ownership			< 0.001			< 0.001		
Non-profit	425	67.0		467	73.5		395	62.2
Govt. non-fed	85	13.4		58	9.1		117	18.4
For-profit	124	19.6		110	17.3		123	19.4
Bed size			< 0.001			< 0.001		
< 99	171	27.0		104	16.5		50.0	7.9
100–199	200	31.6		186	29.3		146	23.0
> =200	263	41.5		344	54.2		439	69.1
Urban/rural location			< 0.001			< 0.001		
Large urban area	247	39.0		311	49.0		468	73.7
Other urban area	300	47.3		272	42.8		159	25.0
Rural area	87	13.7		52	8.2		8	1.3
Disproportionate share hospital			< 0.001			< 0.001		
Low (<= 15%)	225	35.5		108	17		65	10.2
Moderate (15.1 to 35%)	360	56.8		406	63.9		272	42.8
High (35.1%+)	49	7.7		121	19.1		298	46.9
Patient case-mix (hospital average)			< 0.001			< 0.001		
Low complexity tertile	183	28.9		120	18.9		76	12.0
Moderate complexity tertile	266	42.0		237	37.3		195	30.7
High complexity tertile	185	29.2		278	43.8		364	57.3
Region			< 0.001			0.814		
Northeast	120	18.9		132	20.8		120	18.9
Midwest	192	30.3		161	25.4		99	15.6
South	288	45.4		254	40.0		171	26.9
West	34	5.4		88	13.9		245	38.6

1) Hospitals that appear in any of the following samples are included in the table: acute myocardial infarction, heart failure, and pneumonia

2) *p*-values are based on comparisons between high vs. low and moderate vs. low Medicare bed share hospitals; t-test for continuous variables and chi-square test for categorical variables

3) Hospital characteristics are in the baseline year (2009)

The non-parametric relationship between readmissions and the share of Medicare bed days suggests no systematic association between share of Medicare bed days and readmission rates for any of the three conditions – acute myocardial infarction, heart failure, and pneumonia (Additional file 1: Figure A1).

**Fig. 1:**
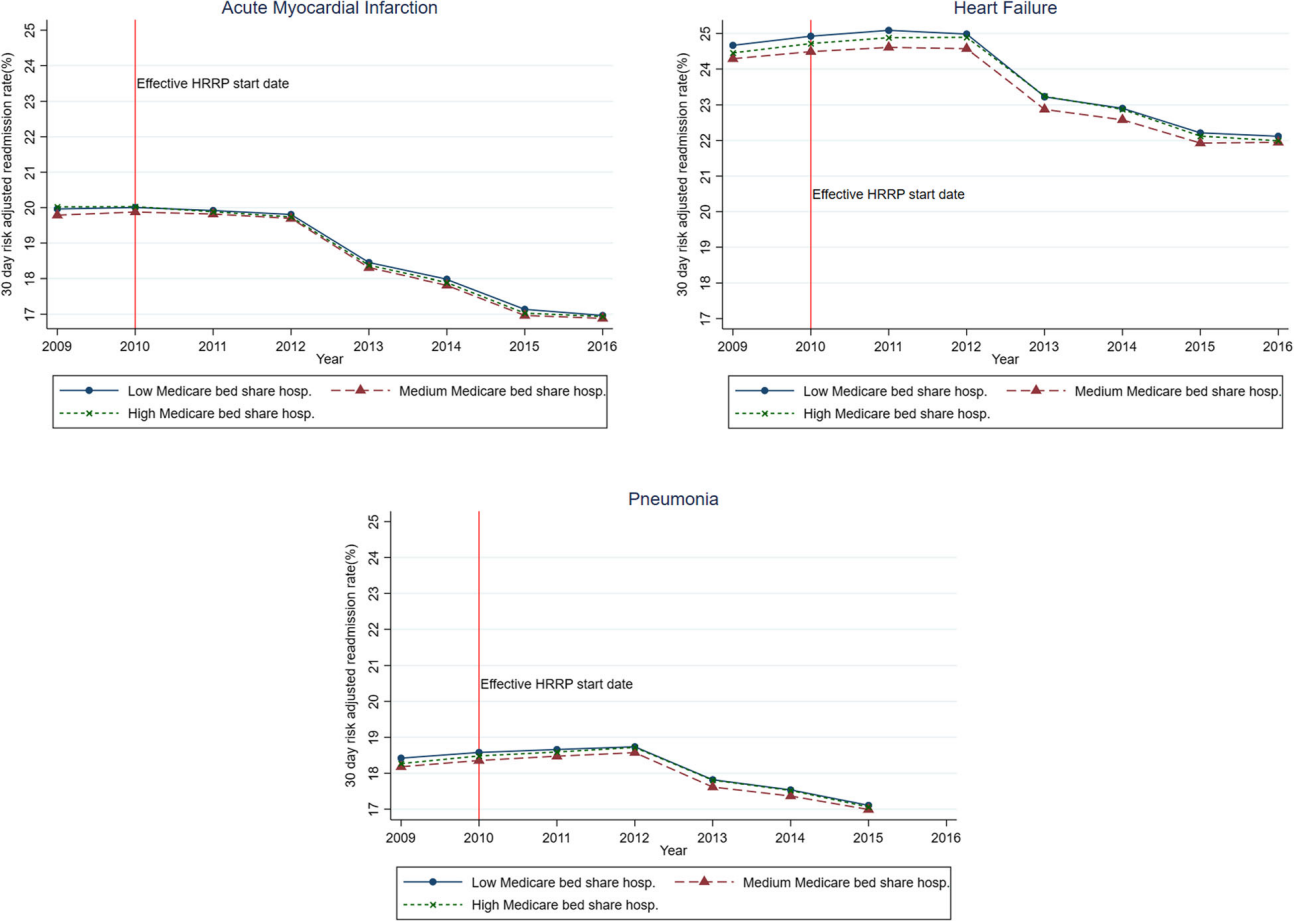
Trends in acute myocardial infarction, heart failure, and pneumonia 30-day risk adjusted readmission rates by Medicare bed share tertiles, 2009–2016. Notes: Effective HRRP start date is 2010 – the year in which the HRRP was enacted as part of the Affordable Care Act. Data sources: Authors’ analysis of Hospital Compare, 2009–2016; American Hospital Association Annual Survey, 2009; and Final Impact Rules, 2009–2016 data

Annual longitudinal trends in risk-adjusted 30-day readmission rates stratified by Medicare bed share tertiles are presented in Fig. 1. Readmission trends were similar between high and low for all the three conditions.

The findings of the association of the HRRP with 30-day readmission rates across hospitals grouped by Medicare bed share using the difference-in-differences approach are shown in Table 2 (full regression results in Table A1 in Additional file 1). In the lowest Medicare bed share hospitals (the reference group), the pre-HRRP rate of 30-day readmissions was 20.0% (AMI), 24.7% (HF) and 18.4% (pneumonia). There was a secular trend towards declining readmission rates from pre- to post-HRRP period for all three tertiles based on Medicare bed share. Among the reference hospitals, the decrease was 1.35% (AMI) 1.02% (HR) and 0.35% (pneumonia).. After adjusting for time trends and baseline differences in hospital characteristics, we found no statistically significant association between HRRP and 30-day readmissions in high and moderate compared to low Medicare bed share hospitals for any of the conditions. As a test of validity of the difference-in-differences approach (“parallel trends test”), we compared pre-HRRP readmission trends – using only 2009–2010 data – between high and low as well as between moderate and low groups and found similar pre-HRRP trends between each comparison group for each condition (Table A2 in Additional file 1).

**Table 2:** Association of 30-day readmissions with Medicare bed share tertiles, 2009–2016

	Average 30-day risk-adjusted readmission rate (%)		
	High Medicare bed share (tertile 3) hospitals	Moderate Medicare bed share hospitals	Low Medicare bed share (tertile 1) hospitals
**A. Acute myocardial infarction**			
Pre-HRRP	20.0	19.8	20.0
Post-HRRP	18.6	18.5	18.6
Pre- to Post-HRRP difference	−1.47***	−1.31***	−1.35***
	[−1.57, −1.36]	[−1.40, −1.21]	[−1.46, −1.24]
Difference-in-differences	−0.11	0.05	Reference
	[−0.26, 0.04]	[−0.10, 0.04]	
**B. Heart failure**			
Pre-HRRP	24.5	24.4	24.7
Post-HRRP	23.5	23.3	23.6
Pre- to Post-HRRP difference	−0.92***	−1.00***	−1.02***
	[−1.05, − 0.79]	[− 1.12, − 0.88]	[−1.15, − 0.90]
Difference-in-differences	0.11	0.03	Reference
	[−0.07, 0.28]	[− 0.07, 0.29]	
**C. Pneumonia**			
Pre-HRRP	18.3	18.2	18.4
Post-HRRP	18.0	17.9	18.1
Pre- to Post-HRRP difference	−0.24***	−0.28***	− 0.35***
	[− 0.34, − 0.13]	[−0.38, − 0.18]	[−0.45, − 0.24]
Difference-in-differences	0.11	0.06	Reference
	[−0.04, 0.26]	[0.08, 0.21]	

1) Pre-HRRP year is 2009; post-HRRP period is 2010–2016

2) Pre- to Post-HRRP differences indicate observed differences within each Medicare bed share cohort between pre- and post-HRRP periods. These estimates are based on linear random effects regression models (separate for each Medicare bed share cohort) with hospital-level clustering. Only covariate included was indicator of Post-HRRP period

3) Difference-in-differences estimates are based on linear random effects regression model. Only the interaction term coefficients are reported above. Full model estimates are in Additional file 1: Table 1

4) 95% confidence intervals are in square parentheses. *** *p* < 0.001

Sensitivity analysis using alternative choice of post-HRRP periods (2011–2016 and 2012–2016) provided largely similar findings of the change in readmission rates associated with hospital Medicare bed share (Additional file 1: Tables A3 and A4). Alternative ways of representing differences in Medicare bed share (as quartiles or as a continuous) did not affect the findings (Additional file 1: Tables A5 and A6). Use of a fixed effects regression specification of hospital-level clustering also yield similar results (Additional file 1: Table A7).

## Discussion

Our findings indicate that the share of Medicare patient bed days out of a hospital’s total bed days, was not associated with changes in 30-day readmissions performance. While there was a secular reduction in readmissions following HRRP, our main analyses found no difference between high vs. low and moderate vs. low Medicare bed share hospitals in the longitudinal reduction in readmissions performance follow HRRP for each of the three admission cohorts (AMI, heart failure and pneumonia). A variety of sensitivity analyses consistently indicated no meaningful differences in change in readmissions associated with Medicare bed share.

Our overall findings are consistent with those from the previous study that did not find any significant association between HRRP program incentives, as measured by Medicare bed share, and changes in readmissions in New York (2008–2013) [12]. Using data on admissions from 144 hospitals for the same age and conditions groups as in our study, that study also found no differences in readmission rates by Medicare bed share in the first two years following HRRP. Our findings of a secular decrease in readmission rates for all three condition cohorts are also consistent with prior studies [9, 11, 33]. We note that the apparent difference in the timing of reductions – beginning in 2012 in our study and 2010 in the prior studies – is a definitional difference, since our readmission rate measure is based on performance in the prior 3-year period (as reported by the CMS Hospital Compare program) while the prior studies report concurrent readmission rates.

In understanding our finding of the absence of an association between Medicare bed share and readmissions performance, one plausible explanation is that the magnitude of penalties may not have been sizable. Several previous studies have noted that HRRP penalties were “small” and unlikely to lead to “meaningful payment differentials” [21, 34, 35]. Although the maximum HRRP penalty ranged from 1% in 2013 to 3% since 2015, the actual average penalty was much smaller. In non-safety net hospitals, the average penalty was 0.28% in 2013 and 0.50% in 2016; even in safety-net hospitals, the average penalty was 0.37% in 2013 and 0.49% in 2016 [36]. Other studies have also indicated that even among safety-net hospitals the financial burden of HRRP has been limited [21, 37].

More generally, across all hospitals, recent evidence seems to suggest that the secular decrease in readmissions following HRRP may be over-estimated [6, 7]. A recent study using national data from 2008 to 2014 found that 60% of the reduction in readmissions was due to change in coding practices – wherein more comorbidities were identified following HRRP leading to lower risk-adjusted readmission rates – rather than actual reductions in readmissions [7, 38, 39]. We note that our findings – based on Hospital Compare methodology that incorporates comorbidities identified in both inpatient and outpatient claims in the prior three years – is less susceptible to changes from coding practices; the prior studies of secular changes in readmissions following HRRP are based only on comorbidities identified in inpatient claims identified in the prior 12 months [9, 33, 38, 39]. In addition to coding, other studies have also raised concerns of other strategic practices – including diverting patients to observation units – to reduce readmission counts [40, 41]. Another study found that while 30-day readmissions decreased following HRRP, ED and outpatient visits within 30-days of incident discharge increased by a larger proportion [6]. Early reduction in 30-day readmissions has also been associated with statistical phenomenon of regression to mean arising from the methodology used in hospital performance measurement [42]. Secular reduction in overall hospitalization rates are also associated with reduction in readmissions [43]. Our study points to considerable variation across hospitals in the share of Medicare patients, and consequently, the reliance on revenues from Medicare payments. For hospitals with higher penalties, there may be an incentive to reduce the reliance on Medicare revenues (and patients). To our knowledge, no study has examined endogenous changes in Medicare patients in response to HRRP penalties.

A number of study limitations need to be noted. First our study includes hospitals that participated in the HRRP and Hospital Compare program in all the study years (2013–2016), and therefore excluded some hospitals that may have closed or reduced intake of Medicare patients. Second, in our analysis, only one year of pre-HRRP data (2009) was available; as a result, we were unable to determine if the pre-HRRP longitudinal trends were similar between high and low Medicare bed share hospitals as well as those between moderate and low (“parallel trends test”) [26]. In supplementary analysis, we re-estimated our difference-in-differences model treating 2009 and 2010 as the pre-HRRP period and 2011–2016 as the post-HRRP period and tested for the similarity of readmission changes between 2009 and 2010 for each condition (“parallel trends test”). The results from these robustness checks were not different from those in the main analysis. Second, public reporting of 30-day hospital readmission rates for acute myocardial infarction, heart failure, and pneumonia by CMS started in 2009; this may have resulted in changes in readmissions for hospitals that were mandated to report their readmissions performance, thereby limiting our ability to disentangle the independent effects of the HRRP-associated changes in readmissions.

## Conclusion

In conclusion, our study findings indicate that the magnitude of the financial burden from HRRP penalties, as captured by a hospital’s Medicare bed share, was not associated with the extent of reduction in 30-day readmissions following HRRP. This is consistent with the broader evidence that the secular reduction in readmissions associated with HRRP may be an over-estimate. It is unclear if the upcoming modifications to HRRP – such as the twenty-first Century Cures Act of 2016, wherein hospital readmission performance will be compared with that of the subgroup of other hospitals with similar proportion of low income patients served – will be more effective in reducing readmissions [44, 45]. As the relative merits of the different modifications are poorly understood, the CMS should also consider working directly and collaboratively – rather than punitively – with hospitals to identify and prioritize quality problems that are most relevant to individual providers, create and support learning systems with hospitals that focus on collecting data for learning and quality improvement, improve transparency around outcomes and providing more financial support for quality improvement efforts at hospitals based on need [46–49].

## Supplementary Information

**Additional file 1: Figure A1.** 30-day readmission rates by share of Medicare bed days, 2009–2016. **Table A1.** Association of HRRP with 30-day readmissions by Medicare bed share tertiles, 2009–2016 - full regression results. **Table A2.** Parallel trends test of association of HRRP with 30-day readmissions by Medicare bed share tertiles with 2009 (“pre”) and 2010 (“post) observations only. **Table A3.** Association of HRRP with 30-day readmissions by Medicare bed share tertiles considering 2011 as HRRP start year, 2009–2016. **Table A4.** Association of HRRP with 30-day readmissions by Medicare bed share tertiles considering 2012 as HRRP start year, 2009–2016. **Table A5.** Association of HRRP with 30-day readmissions using a continuous measure for Medicare bed share, 2009–2016. **Table A6.** Association of HRRP with 30-day readmissions by Medicare bed share quartiles, 2009–2016. **Table A7.** Association of HRRP with 30-day readmissions by Medicare bed share tertiles, 2009–2016 - Hospital fixed effects specification.

## Data Availability

We used publicly available nationwide hospital level data for 2009–2016 from the Centers for Medicare and Medicaid Services (CMS) Hospital Compare program http://www.medicare.gov/hospitalcompare/search.html;2018, CMS Final Impact Rule https://www.cms.gov/Medicare/Medicare-Fee-for-Service-Payment/AcuteInpatientPPS/Historical-Impact-Files-for-FY-1994-through-Present.html;2017 and the American Hospital Association Annual Survey (www.ahadata.com).

## Abbreviations

HRRP: Medicare’s Hospital Readmissions Reduction Program
IPPS: Inpatient Prospective Payment System
CMS: Centers for Medicare and Medicaid Services
AMI: Acute Myocardial Infarction
HF: Heart Failure
