# Supplementary material for: Association between Medicare’s Hospital Readmission Reduction Program and readmission rates across hospitals by medicare bed share
Source: BMC Health Serv Res. 2021 Mar 19;21:248. doi: 10.1186/s12913-021-06253-2 (PMC7980319; doi:10.1186/s12913-021-06253-2)
Supplement: Supplementary file 1 — Additional file 1: Figure A1. 30-day readmission rates by share of Medicare bed days, 2009–2016. Table A1. Association of HRRP with 30-day readmissions by Medicare bed share tertiles, 2009–2016 - full regression results. Table A2. Parallel trends test of association of HRRP with 30-day readmissions by Medicare bed share tertiles with 2009 (“pre”) and 2010 (“post) observations only. Table A3. Association of HRRP with 30-day readmissions by Medicare bed share tertiles considering 2011 as HRRP start year, 2009–2016. Table A4. Association of HRRP with 30-day readmissions by Medicare bed share tertiles considering 2012 as HRRP start year, 2009–2016. Table A5. Association of HRRP with 30-day readmissions using a continuous measure for Medicare bed share, 2009–2016. Table A6. Association of HRRP with 30-day readmissions by Medicare bed share quartiles, 2009–2016. Table A7. Association of HRRP with 30-day readmissions by Medicare bed share tertiles, 2009–2016 - Hospital fixed effects specification. [file 12913_2021_6253_MOESM1_ESM.docx]

**Appendix**

**Figure A1: 30-day readmission rates by share of Medicare bed days, 2009-2016**


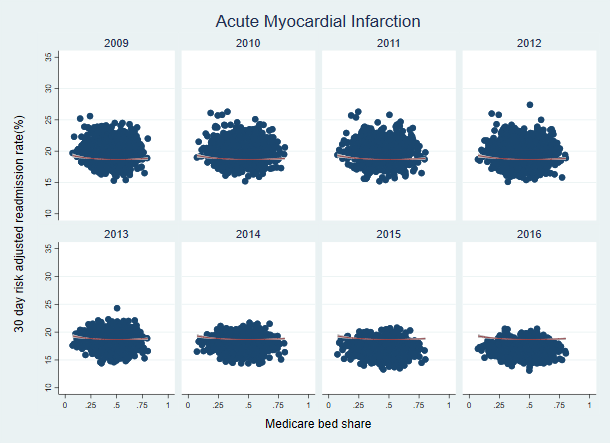

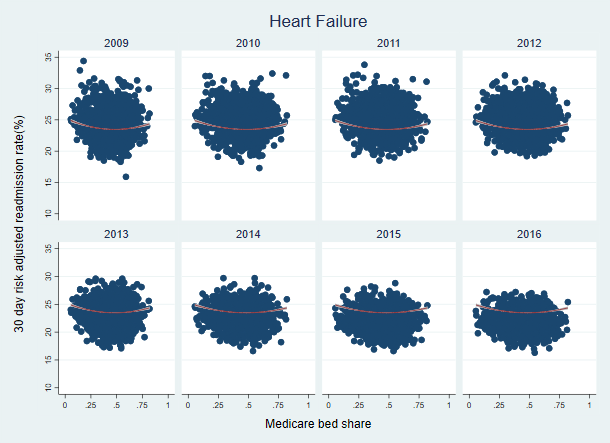


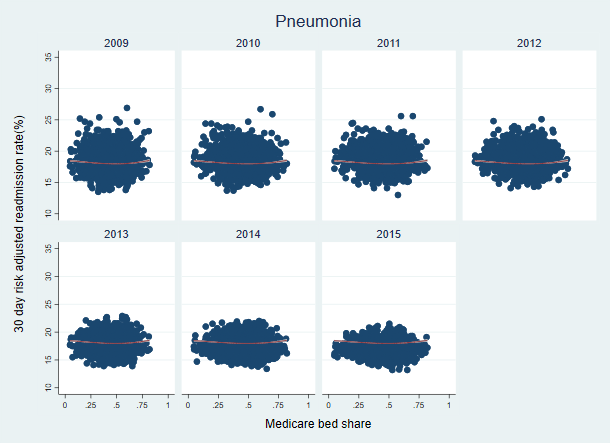


Notes: Each dot represents one acute care hospital. The trend line was estimated from a fractional polynomial regression model of readmissions as a function of the share of Medicare bed days.

Table A1: Association of HRRP with 30-day readmissions by Medicare bed share tertiles, 2009-2016 - full regression results

|  | Acute Myocardial Infarction | Heart Failure | Pneumonia |
| --- | --- | --- | --- |
| Medicare bed share: Moderate (ref: Low) | 0.024 | -0.036 | -0.029 |
|  | [-0.156,0.204] | [-0.261,0.189] | [-0.214,0.156] |
| High | 0.370*** | 0.300* | 0.209* |
|  | [0.169,0.571] | [0.056,0.543] | [0.007,0.411] |
| Post-HRRP | -2.972*** | -2.495*** | -1.295*** |
|  | [-3.092,-2.852] | [-2.636,-2.354] | [-1.411,-1.179] |
| Moderate Medicare bed share x Post-HRRP | 0.046 | 0.025 | 0.063 |
|  | [-0.100,0.192] | [-0.147,0.198] | [-0.080,0.206] |
| High Medicare bed share x Post-HRRP | -0.114 | 0.107 | 0.109 |
|  | [-0.266,0.037] | [-0.072,0.286] | [-0.037,0.255] |
| Teaching hospitals | 0.289*** | 0.446*** | 0.481*** |
|  | [0.121,0.457] | [0.220,0.671] | [0.296,0.667] |
| Hospital ownership: Government (ref: Non-profit) | 0.039 | 0.1 | 0.012 |
|  | [-0.119,0.197] | [-0.095,0.295] | [-0.148,0.172] |
| For-profit | 0.192** | 0.561*** | 0.254*** |
|  | [0.064,0.321] | [0.396,0.725] | [0.120,0.387] |
| Bed size: 100 to 199 (ref: < 100) | 0.116 | 0.257** | 0.263*** |
|  | [-0.059,0.292] | [0.067,0.448] | [0.112,0.414] |
| 200 and more | 0.168 | 0.402*** | 0.400*** |
|  | [-0.019,0.356] | [0.197,0.607] | [0.236,0.563] |
| Urban areas: Other urban (ref: Large urban) | -0.435*** | -0.724*** | -0.544*** |
|  | [-0.539,-0.332] | [-0.860,-0.587] | [-0.653,-0.435] |
| Rural | -0.411** | -0.575*** | -0.514*** |
|  | [-0.713,-0.110] | [-0.849,-0.302] | [-0.727,-0.302] |
| Disproportionate share hospital: Moderate (ref: Low) | 0.303*** | 0.563*** | 0.375*** |
|  | [0.184,0.422] | [0.405,0.721] | [0.244,0.506] |
| High | 0.765*** | 1.337*** | 0.743*** |
|  | [0.606,0.924] | [1.123,1.551] | [0.565,0.921] |
| Hospital case mix: Moderate (ref: Low) | -0.011 | -0.237* | -0.198* |
|  | [-0.167,0.144] | [-0.429,-0.044] | [-0.351,-0.045] |
| High complexity | -0.238* | -0.903*** | -0.430*** |
|  | [-0.419,-0.056] | [-1.129,-0.676] | [-0.604,-0.256] |
| Region: Midwest (ref: Northeast) | -0.296*** | -0.674*** | -0.294*** |
|  | [-0.440,-0.153] | [-0.868,-0.479] | [-0.451,-0.137] |
| South | -0.501*** | -0.805*** | -0.610*** |
|  | [-0.646,-0.355] | [-1.002,-0.608] | [-0.769,-0.451] |
| West | -0.791*** | -1.176*** | -0.956*** |
|  | [-0.956,-0.627] | [-1.398,-0.954] | [-1.131,-0.782] |
| Reference cohort (Intercept) | 19.950*** | 24.769*** | 18.429*** |
|  | [19.681,20.219] | [24.478,25.061] | [18.190,18.669] |
| N | 11520 | 14928 | 13188 |

Notes:

1) 95% confidence intervals are in square parentheses. Estimates from random effects model reported; heteroscedasticity-robust standard errors clustered at the hospital level. * p<0.05; ** p<0.01; *** p<0.001

2) Pre-HRRP year is 2009; post-HRRP period is 2010-2016 (AMI and HF) and 2010-2015 (pneumonia).

3) The regression model also includes year indicators, that are not reported above.

Table A2: Parallel trends test of association of HRRP with 30-day readmissions by Medicare bed share tertiles with 2009 ("pre") and 2010 ("post) observations only

|  | Acute Myocardial Infarction | Heart Failure | Pneumonia |
| --- | --- | --- | --- |
| Medicare bed share: Moderate (ref: Low) | 0.039 | -0.006 | 0.057 |
|  | [-0.152,0.230] | [-0.238,0.227] | [-0.136,0.251] |
| High | 0.368** | 0.305* | 0.326** |
|  | [0.144,0.591] | [0.045,0.565] | [0.107,0.545] |
| Post-HRRP | 0.043 | 0.257*** | 0.159*** |
|  | [-0.041,0.126] | [0.168,0.346] | [0.085,0.233] |
| Moderate Medicare bed share x Post-HRRP | 0.045 | -0.058 | 0.017 |
|  | [-0.066,0.156] | [-0.182,0.066] | [-0.086,0.120] |
| High Medicare bed share x Post-HRRP | -0.037 | 0.005 | 0.054 |
|  | [-0.151,0.077] | [-0.121,0.131] | [-0.050,0.159] |
| Teaching hospitals | 0.428*** | 0.576*** | 0.627*** |
|  | [0.195,0.660] | [0.279,0.873] | [0.382,0.872] |
| Hospital ownership: Government (ref: Non-profit) | 0.116 | 0.315* | 0.197 |
|  | [-0.115,0.346] | [0.048,0.581] | [-0.027,0.421] |
| For-profit | 0.094 | 0.626*** | 0.306** |
|  | [-0.077,0.265] | [0.389,0.862] | [0.119,0.493] |
| Bed size: 100 to 199 (ref: < 100) | 0.063 | 0.375** | 0.270* |
|  | [-0.198,0.324] | [0.090,0.660] | [0.050,0.490] |
| 200 and more | 0.179 | 0.471** | 0.370** |
|  | [-0.098,0.457] | [0.155,0.787] | [0.128,0.612] |
| Urban areas: Other urban (ref: Large urban) | -0.536*** | -0.902*** | -0.680*** |
|  | [-0.677,-0.394] | [-1.086,-0.718] | [-0.827,-0.532] |
| Rural | -0.24 | -0.809*** | -0.609*** |
|  | [-0.666,0.187] | [-1.200,-0.418] | [-0.912,-0.306] |
| Disproportionate share hospitals: Moderate (ref: Low) | 0.347*** | 0.565*** | 0.425*** |
|  | [0.176,0.519] | [0.346,0.784] | [0.249,0.600] |
| High | 0.830*** | 1.340*** | 0.915*** |
|  | [0.601,1.060] | [1.051,1.630] | [0.669,1.161] |
| Hospital case mix: Moderate (ref: Low) | -0.043 | -0.409** | -0.317** |
|  | [-0.274,0.188] | [-0.684,-0.135] | [-0.542,-0.093] |
| High complexity | -0.456*** | -1.263*** | -0.483*** |
|  | [-0.717,-0.195] | [-1.590,-0.937] | [-0.736,-0.229] |
| Region: Midwest (ref: Northeast) | -0.256* | -0.504*** | -0.504*** |
|  | [-0.464,-0.049] | [-0.768,-0.241] | [-0.724,-0.285] |
| South | -0.737*** | -0.930*** | -0.973*** |
|  | [-0.937,-0.538] | [-1.189,-0.671] | [-1.189,-0.756] |
| West | -0.995*** | -1.285*** | -1.307*** |
|  | [-1.226,-0.764] | [-1.578,-0.991] | [-1.553,-1.061] |
| Reference cohort (Intercept) | 20.182*** | 24.963*** | 18.633*** |
|  | [19.808,20.555] | [24.586,25.340] | [18.314,18.953] |
| N | 2880 | 3732 | 3768 |

Notes:

1) 95% confidence intervals are in square parentheses. Estimates from random effects model reported; heteroscedasticity-robust standard errors clustered at the hospital level. * p<0.05; ** p<0.01; *** p<0.001

2) Pre-HRRP year is 2009; post-HRRP period is 2010.

3) The regression model also includes year indicators, that are not reported above.

Table A3: Association of HRRP with 30-day readmissions by Medicare bed share tertiles considering 2011 as HRRP start year, 2009-2016

|  | Acute Myocardial Infarction | Heart Failure | Pneumonia |
| --- | --- | --- | --- |
| Medicare bed share: Moderate (ref: Low) | 0.047 | -0.065 | -0.021 |
|  | [-0.121,0.215] | [-0.275,0.146] | [-0.195,0.153] |
| High | 0.352*** | 0.302* | 0.236* |
|  | [0.161,0.542] | [0.072,0.533] | [0.045,0.427] |
| Post-HRRP | -2.966*** | -2.514*** | -1.290*** |
|  | [-3.084,-2.849] | [-2.651,-2.376] | [-1.405,-1.175] |
| Moderate Medicare bed share x Post-HRRP | 0.023 | 0.069 | 0.064 |
|  | [-0.117,0.163] | [-0.098,0.235] | [-0.076,0.204] |
| High Medicare bed share x Post-HRRP | -0.109 | 0.122 | 0.093 |
|  | [-0.255,0.038] | [-0.051,0.294] | [-0.051,0.236] |
| Teaching hospitals | 0.289*** | 0.446*** | 0.481*** |
|  | [0.121,0.457] | [0.220,0.671] | [0.296,0.667] |
| Hospital ownership: Government (ref: Non-profit) | 0.039 | 0.1 | 0.012 |
|  | [-0.119,0.197] | [-0.095,0.295] | [-0.148,0.172] |
| For-profit | 0.192** | 0.561*** | 0.254*** |
|  | [0.064,0.321] | [0.396,0.725] | [0.120,0.387] |
| Bed size: 100 to 199 (ref: < 100) | 0.116 | 0.257** | 0.263*** |
|  | [-0.059,0.292] | [0.067,0.448] | [0.112,0.414] |
| 200 and more | 0.168 | 0.402*** | 0.400*** |
|  | [-0.019,0.356] | [0.197,0.607] | [0.236,0.563] |
| Urban areas: Other urban (ref: Large urban) | -0.435*** | -0.724*** | -0.544*** |
|  | [-0.539,-0.332] | [-0.860,-0.587] | [-0.653,-0.435] |
| Rural | -0.411** | -0.575*** | -0.514*** |
|  | [-0.713,-0.110] | [-0.849,-0.302] | [-0.727,-0.302] |
| Disproportionate share hospital: Moderate (ref: Low) | 0.303*** | 0.563*** | 0.375*** |
|  | [0.184,0.422] | [0.405,0.721] | [0.244,0.506] |
| High | 0.765*** | 1.337*** | 0.743*** |
|  | [0.606,0.924] | [1.123,1.551] | [0.565,0.921] |
| Hospital case mix: Moderate (ref: Low) | -0.011 | -0.237* | -0.198* |
|  | [-0.167,0.144] | [-0.429,-0.044] | [-0.351,-0.045] |
| High complexity | -0.238* | -0.903*** | -0.430*** |
|  | [-0.419,-0.056] | [-1.129,-0.676] | [-0.604,-0.256] |
| Region: Midwest (ref: Northeast) | -0.296*** | -0.674*** | -0.294*** |
|  | [-0.440,-0.153] | [-0.868,-0.479] | [-0.451,-0.137] |
| South | -0.501*** | -0.805*** | -0.610*** |
|  | [-0.646,-0.355] | [-1.002,-0.608] | [-0.769,-0.451] |
| West | -0.791*** | -1.176*** | -0.956*** |
|  | [-0.956,-0.627] | [-1.398,-0.954] | [-1.131,-0.782] |
| Reference cohort (Intercept) | 19.948*** | 24.778*** | 18.418*** |
|  | [19.681,20.215] | [24.491,25.066] | [18.180,18.655] |
| N | 11520 | 14928 | 13188 |

Notes:

1) 95% confidence intervals are in square parentheses. Estimates from random effects model reported; heteroscedasticity-robust standard errors clustered at the hospital level. * p<0.05; ** p<0.01; *** p<0.001

2) Pre-HRRP year is 2009-2010; post-HRRP period is 2011-2016 (AMI and HF) and 2011-2015 (pneumonia).

3) The regression model also includes year indicators, that are not reported above.

Table A4: Association of HRRP with 30-day readmissions by Medicare bed share tertiles considering 2012 as HRRP start year, 2009-2016

|  | Acute Myocardial Infarction | Heart Failure | Pneumonia |
| --- | --- | --- | --- |
| Medicare bed share: Moderate (ref: Low) | 0.064 | -0.089 | -0.006 |
|  | [-0.097,0.225] | [-0.291,0.113] | [-0.168,0.156] |
| High | 0.326*** | 0.304** | 0.255** |
|  | [0.143,0.510] | [0.083,0.525] | [0.073,0.436] |
| Post-HRRP | -2.964*** | -2.539*** | -1.283*** |
|  | [-3.079,-2.849] | [-2.675,-2.403] | [-1.397,-1.170] |
| Moderate Medicare bed share x Post-HRRP | 0 | 0.121 | 0.053 |
|  | [-0.137,0.137] | [-0.043,0.286] | [-0.082,0.189] |
| High Medicare bed share x Post-HRRP | -0.089 | 0.144 | 0.083 |
|  | [-0.234,0.055] | [-0.026,0.313] | [-0.056,0.223] |
| Teaching hospitals | 0.289*** | 0.446*** | 0.481*** |
|  | [0.121,0.457] | [0.220,0.671] | [0.296,0.667] |
| Hospital ownership: Government (ref: Non-profit) | 0.039 | 0.1 | 0.012 |
|  | [-0.119,0.197] | [-0.095,0.295] | [-0.148,0.172] |
| For-profit | 0.192** | 0.561*** | 0.254*** |
|  | [0.064,0.321] | [0.396,0.725] | [0.120,0.387] |
| Bed size: 100 to 199 (ref: < 100) | 0.116 | 0.257** | 0.263*** |
|  | [-0.059,0.292] | [0.067,0.448] | [0.112,0.414] |
| 200 and more | 0.168 | 0.402*** | 0.400*** |
|  | [-0.019,0.356] | [0.197,0.607] | [0.236,0.563] |
| Urban areas: Other urban (ref: Large urban) | -0.435*** | -0.724*** | -0.544*** |
|  | [-0.539,-0.332] | [-0.860,-0.587] | [-0.653,-0.435] |
| Rural | -0.411** | -0.575*** | -0.514*** |
|  | [-0.713,-0.110] | [-0.849,-0.302] | [-0.727,-0.302] |
| Disproportionate share hospital: Moderate (ref: Low) | 0.303*** | 0.563*** | 0.375*** |
|  | [0.184,0.422] | [0.405,0.721] | [0.244,0.506] |
| High | 0.765*** | 1.337*** | 0.743*** |
|  | [0.606,0.924] | [1.123,1.551] | [0.565,0.921] |
| Hospital case mix: Moderate (ref: Low) | -0.011 | -0.237* | -0.198* |
|  | [-0.167,0.144] | [-0.429,-0.044] | [-0.351,-0.045] |
| High complexity | -0.238* | -0.903*** | -0.430*** |
|  | [-0.419,-0.056] | [-1.129,-0.676] | [-0.604,-0.256] |
| Region: Midwest (ref: Northeast) | -0.296*** | -0.674*** | -0.294*** |
|  | [-0.440,-0.153] | [-0.868,-0.479] | [-0.451,-0.137] |
| South | -0.501*** | -0.805*** | -0.610*** |
|  | [-0.646,-0.355] | [-1.002,-0.608] | [-0.769,-0.451] |
| West | -0.791*** | -1.176*** | -0.956*** |
|  | [-0.956,-0.627] | [-1.398,-0.954] | [-1.131,-0.782] |
| Reference cohort (Intercept) | 19.950*** | 24.786*** | 18.406*** |
|  | [19.685,20.215] | [24.500,25.072] | [18.172,18.641] |
| N | 11520 | 14928 | 13188 |

Notes:

1) 95% confidence intervals are in square parentheses. Estimates from random effects model reported; heteroscedasticity-robust standard errors clustered at the hospital level. * p<0.05; ** p<0.01; *** p<0.001

2) Pre-HRRP year is 2009-2011; post-HRRP period is 2012-2016 (AMI and HF) and 2012-2015 (pneumonia).

3) The regression model also includes year indicators, that are not reported above.

Table A5: Association of HRRP with 30-day readmissions using a continuous measure for Medicare bed share, 2009-2016

|  | Acute Myocardial Infarction | Heart Failure | Pneumonia |
| --- | --- | --- | --- |
| Medicare bed share | 1.019** | 1.082** | 0.702* |
|  | [0.337,1.701] | [0.288,1.875] | [0.042,1.362] |
| Post-HRRP | -2.896*** | -2.543*** | -1.442*** |
|  | [-3.144,-2.649] | [-2.826,-2.261] | [-1.668,-1.215] |
| Medicare bed share x Post-HRRP | -0.208 | 0.201 | 0.441 |
|  | [-0.711,0.296] | [-0.376,0.777] | [-0.023,0.904] |
| Teaching hospitals | 0.294*** | 0.474*** | 0.507*** |
|  | [0.125,0.463] | [0.244,0.704] | [0.319,0.695] |
| Hospital ownership: Government (ref: Non-profit) | 0.066 | 0.165 | 0.058 |
|  | [-0.092,0.225] | [-0.029,0.359] | [-0.102,0.217] |
| For-profit | 0.195** | 0.573*** | 0.262*** |
|  | [0.066,0.324] | [0.408,0.737] | [0.129,0.395] |
| Bed size: 100 to 199 (ref: < 100) | 0.112 | 0.259** | 0.268*** |
|  | [-0.062,0.287] | [0.068,0.450] | [0.118,0.418] |
| 200 and more | 0.167 | 0.413*** | 0.409*** |
|  | [-0.020,0.354] | [0.208,0.619] | [0.246,0.573] |
| Urban areas: Other urban (ref: Large urban) | -0.439*** | -0.735*** | -0.556*** |
|  | [-0.543,-0.335] | [-0.872,-0.597] | [-0.666,-0.446] |
| Rural | -0.392* | -0.570*** | -0.514*** |
|  | [-0.692,-0.091] | [-0.845,-0.295] | [-0.725,-0.303] |
| Disproportionate share hospital: Moderate (ref: Low) | 0.292*** | 0.535*** | 0.363*** |
|  | [0.173,0.411] | [0.379,0.691] | [0.234,0.492] |
| High | 0.771*** | 1.341*** | 0.763*** |
|  | [0.609,0.932] | [1.123,1.559] | [0.581,0.944] |
| Hospital case mix: Moderate (ref: Low) | -0.008 | -0.233* | -0.190* |
|  | [-0.163,0.147] | [-0.426,-0.040] | [-0.343,-0.038] |
| High complexity | -0.236* | -0.911*** | -0.428*** |
|  | [-0.418,-0.053] | [-1.138,-0.685] | [-0.603,-0.254] |
| Region: Midwest (ref: Northeast) | -0.289*** | -0.664*** | -0.291*** |
|  | [-0.431,-0.146] | [-0.859,-0.470] | [-0.447,-0.134] |
| South | -0.503*** | -0.799*** | -0.609*** |
|  | [-0.649,-0.357] | [-0.996,-0.601] | [-0.769,-0.450] |
| West | -0.782*** | -1.138*** | -0.923*** |
|  | [-0.949,-0.616] | [-1.362,-0.913] | [-1.099,-0.746] |
| Reference cohort (Intercept) | 19.603*** | 24.343*** | 18.142*** |
|  | [19.179,20.028] | [23.867,24.820] | [17.751,18.532] |
| N | 11520 | 14928 | 13188 |

Notes:

1) 95% confidence intervals are in square parentheses. Estimates from random effects model reported; heteroscedasticity-robust standard errors clustered at the hospital level. * p<0.05; ** p<0.01; *** p<0.001

2) Pre-HRRP year is 2009; post-HRRP period is 2010-2016 (AMI and HF) and 2010-2015 (pneumonia).

3) The regression model also includes year indicators, that are not reported above.

Table A6: Association of HRRP with 30-day readmissions by Medicare bed share quartiles, 2009-2016

|  | Acute Myocardial Infarction | Heart Failure | Pneumonia |
| --- | --- | --- | --- |
| Medicare bed share: Quartile 2 (ref: Lowest share quartile) | -0.093 | -0.243 | -0.161 |
|  | [-0.301,0.115] | [-0.499,0.013] | [-0.374,0.051] |
| Quartile 3 | 0.105 | 0.234 | 0.036 |
|  | [-0.109,0.318] | [-0.036,0.505] | [-0.182,0.254] |
| Quartile 4 | 0.363** | 0.388** | 0.241* |
|  | [0.130,0.597] | [0.105,0.672] | [0.008,0.475] |
| Post-HRRP | -2.971*** | -2.512*** | -1.334*** |
|  | [-3.106,-2.835] | [-2.675,-2.350] | [-1.463,-1.205] |
| Quartile 2 Medicare bed share x Post-HRRP | 0.033 | 0.148 | 0.095 |
|  | [-0.140,0.206] | [-0.050,0.346] | [-0.071,0.261] |
| Quartile 3 Medicare bed share x Post-HRRP | -0.016 | 0.043 | 0.15 |
|  | [-0.186,0.155] | [-0.163,0.250] | [-0.016,0.315] |
| Quartile 4 Medicare bed share x Post-HRRP | -0.115 | 0.055 | 0.14 |
|  | [-0.290,0.060] | [-0.157,0.266] | [-0.029,0.309] |
| Teaching hospitals | 0.283*** | 0.475*** | 0.497*** |
|  | [0.115,0.451] | [0.246,0.703] | [0.309,0.684] |
| Hospital ownership: Government (ref: Non-profit) | 0.028 | 0.1 | 0.005 |
|  | [-0.129,0.185] | [-0.093,0.293] | [-0.154,0.163] |
| For-profit | 0.181** | 0.553*** | 0.246*** |
|  | [0.052,0.310] | [0.388,0.718] | [0.113,0.379] |
| Bed size: 100 to 199 (ref: < 100) | 0.123 | 0.270** | 0.273*** |
|  | [-0.054,0.301] | [0.080,0.461] | [0.123,0.423] |
| 200 and more | 0.173 | 0.411*** | 0.406*** |
|  | [-0.016,0.362] | [0.206,0.615] | [0.243,0.568] |
| Urban areas: Other urban (ref: Large urban) | -0.432*** | -0.739*** | -0.555*** |
|  | [-0.535,-0.328] | [-0.875,-0.603] | [-0.665,-0.446] |
| Rural | -0.426** | -0.580*** | -0.509*** |
|  | [-0.725,-0.127] | [-0.856,-0.304] | [-0.723,-0.295] |
| Disproportionate share hospital: Moderate (ref: Low) | 0.311*** | 0.563*** | 0.387*** |
|  | [0.191,0.431] | [0.404,0.722] | [0.256,0.519] |
| High | 0.766*** | 1.360*** | 0.767*** |
|  | [0.604,0.928] | [1.144,1.575] | [0.587,0.947] |
| Hospital case mix: Moderate (ref: Low) | -0.014 | -0.227* | -0.185* |
|  | [-0.168,0.140] | [-0.419,-0.035] | [-0.338,-0.031] |
| High complexity | -0.238** | -0.883*** | -0.406*** |
|  | [-0.419,-0.057] | [-1.109,-0.656] | [-0.581,-0.231] |
| Region: Midwest (ref: Northeast) | -0.283*** | -0.662*** | -0.288*** |
|  | [-0.425,-0.141] | [-0.855,-0.469] | [-0.444,-0.133] |
| South | -0.498*** | -0.802*** | -0.611*** |
|  | [-0.643,-0.353] | [-0.998,-0.606] | [-0.769,-0.453] |
| West | -0.798*** | -1.141*** | -0.938*** |
|  | [-0.962,-0.634] | [-1.364,-0.918] | [-1.114,-0.762] |
| Reference cohort (Intercept) | 19.981*** | 24.730*** | 18.426*** |
|  | [19.693,20.268] | [24.413,25.047] | [18.168,18.685] |
| N | 11520 | 14928 | 13188 |

Notes:

1) 95% confidence intervals are in square parentheses. Estimates from random effects model reported; heteroscedasticity-robust standard errors clustered at the hospital level. * p<0.05; ** p<0.01; *** p<0.001

2) Pre-HRRP year is 2009; post-HRRP period is 2010-2016 (AMI and HF) and 2010-2015 (pneumonia).

3) The regression model also includes year indicators, that are not reported above.

Table A7: Association of HRRP with 30-day readmissions by Medicare bed share tertiles, 2009-2016 - Hospital fixed effects specification

|  | Acute Myocardial Infarction | Heart Failure | Pneumonia |
| --- | --- | --- | --- |
| Post-HRRP | -2.972*** | -2.495*** | -1.295*** |
|  | [-3.092,-2.852] | [-2.636,-2.354] | [-1.411,-1.179] |
| Moderate Medicare bed share x Post-HRRP | 0.046 | 0.025 | 0.063 |
|  | [-0.100,0.192] | [-0.147,0.198] | [-0.080,0.206] |
| High Medicare bed share x Post-HRRP | -0.114 | 0.107 | 0.109 |
|  | [-0.266,0.037] | [-0.072,0.286] | [-0.037,0.255] |
| Reference cohort (Intercept) | 19.919*** | 24.469*** | 18.289*** |
|  | [19.867,19.971] | [24.406,24.532] | [18.239,18.340] |
| N | 11520 | 14928 | 13188 |

Notes:

1) 95% confidence intervals are in square parentheses. Estimates from hospital-level fixed effects model reported; heteroscedasticity-robust standard errors clustered at the hospital level. * p<0.05; ** p<0.01; *** p<0.001

2) Pre-HRRP year is 2009; post-HRRP period is 2010-2016 (AMI and HF) and 2010-2015 (pneumonia).

3) The regression model also includes year indicators, that are not reported above. Note that the hospital-level fixed effects specification, by design, excludes all covariates that are constant at the hospital level; this results in the exclusion of hospital and region-level covariates.
